# Physiological and antioxidant responses of marjoram (*Origanum Majorana* L.) under drought stress mediated by Salicylic acid and mycorrhizal symbiosis

**DOI:** 10.1186/s12870-025-07225-y

**Published:** 2025-09-30

**Authors:** Babak Modara, Mohammad Mehdi Rahimi, Moslem Abdipour, Mehdi Hosseinifarahi

**Affiliations:** 1 Department of Agrotechnology, Yas. C, Islamic Azad University, Yasuj, Iran; 2 Kohgiluyeh and Boyerahmad Agricultural and Natural Resources Research and Education Center, Agricultural Research, Education and Extension Organization (AREEO), Yasuj, Iran; 3 Department of Horticultural Science, Yas. C., Islamic Azad University, Yasuj, Iran

**Keywords:** Antioxidant enzymes, Climate change adaptation, Factor analysis, *Glomus Hoi*, Plant stress tolerance

## Abstract

**Abstract:**

Drought stress, exacerbated by climate change, is a major limiting factor for herbs cultivation. This study aimed to evaluate the combined effects of salicylic acid (SA) and mycorrhizal fungi (MF) on marjoram under drought stress conditions. The experiment was conducted over two years (2022–2023) using a split factorial design within a randomized complete block with three replications. The study’s primary factor was drought stress at three levels: 90% (D0), 70% (D1), and 35% (D2) of field capacity (FC). The secondary factor included two sub-factors: SA concentrations (0, 100, and 300 mg L^−1^) and MF inoculation (non-inoculated (M0) and inoculated with *Glomus hoi* (M1)). Results demonstrated that drought stress decreased relative water content (RWC) (46.8%), chlorophyll content (35%), carotenoids (25.7%), and dry weight (49.3%), while increasing proline (38.6%), soluble sugars (29.4%), electrolyte leakage (44.8%), superoxide dismutase (35.2%), peroxidase (43.1%), and catalase activities (29.3%). Additionally, the combined treatment of SA and MF enhanced water status by 44%, proline content by 12%, and soluble sugar content by 6% under severe drought conditions. Antioxidant enzyme activities (Catalase) were also significantly increased by up to 91% with the combined treatments, supporting the hypothesis that the synergy of SA and MF can effectively mitigate the adverse effects of drought stress on marjoram. Overall, this study demonstrated that the combined application of SA and MF could be a promising strategy for enhancing drought tolerance in marjoram, especially in drought-prone areas.

**Trial registration:**

This study does not involve clinical trials or human participants and, as such, does not require clinical trial registration.

**Supplementary Information:**

The online version contains supplementary material available at 10.1186/s12870-025-07225-y.

## Introduction

Marjoram (*Origanum majorana* L.) is a widely known aromatic herb that has been cultivated in Mediterranean regions since ancient times [1]. Its popularity grew during the Middle Ages, due to its versatile uses both as a medicinal plant and a flavoring agent [1]. Furthermore, drought stress has emerged as a critical environmental factor that significantly hampers crop production and sustainability, especially in medicinal plants, on a global scale [2]. This issue is further aggravated by climate change, which directly affects the allocation of photo-assimilates and disrupts a range of physiological, biochemical, and molecular processes in plants [3]. In other words, drought stress disrupts multiple plant processes, including water and nutrient uptake, photosynthesis, and the allocation of photo-assimilates, leading to impaired growth and development [4–6]. One of the key consequences of drought is the overproduction of reactive oxygen species (ROS), which leads to widespread cellular damage and impairs various biochemical and molecular functions, causing metabolic activity disruption [6]. To mitigate ROS-induced damage, plants activate a unique defense mechanism and maintain redox balance, thereby protecting physiological and biochemical pathways from further damage [7]. This antioxidant defense system includes key enzymes such as superoxide dismutase (SOD), catalase (CAT), and peroxidase (POD) [3]. However, these endogenous anti-drought compounds are often insufficient to enable crops to withstand prolonged drought conditions. Therefore, the external application of stress tolerance-inducing substances becomes essential for effectively enhancing drought tolerance in plants. In addition to drought, other abiotic stresses, such as chilling stress and temperature extremes disrupt cellular homeostasis and impose oxidative stress. Recent studies, such as [8] and [9] have elucidated pathways through which abiotic stresses trigger the upregulation of Heat Shock Proteins (Hsps) and assimilate redistribution to enhance plant stress tolerance. These findings highlight the importance of integrative approaches to improve plant adaptation to environmental challenges.

Salicylic acid (SA) is a naturally occurring plant hormone that plays diverse roles in regulating various physiological and biochemical processes related to plant growth, including cell division, stomatal closure, photosynthesis, transpiration, nutrient uptake, enzyme activity, and antioxidant defense [10]. Additionally, SA serves as an endogenous signaling molecule, enhancing plant resistance to environmental stresses such as drought [7], salinity [11], and cold [12]. SA treatments have been shown to increase antioxidant enzyme activity, reduce ROS levels, and regulate proline metabolism in *Origanum vulgare* under stress conditions [6]. Besides, Abdali [7] exposed oregano plants to several stress modulators, including SA and four other compounds at three levels of irrigation regimes (40, 60, and 75% field capacity (FC)). They found that these stress modulator compounds improved plants’ morphological traits, relative water content (RWC), and photosynthetic compounds.

Among contemporary eco-friendly technologies in sustainable agriculture, the use of mycorrhizal fungi (MF) is particularly promising, especially for cultivating medicinal plants [13, 14]. These symbiotic fungi enhance plant growth and water absorption by expanding the root system, thereby mitigating water stress [15]. Recent studies emphasize the crucial role of MF in improving plant resilience under drought conditions, as they increase nutrient and water uptake, enhance the antioxidant defense system, and reduce oxidative stress [16]. Particularly, Li [17] revealed that arbuscular mycorrhizal fungi inoculation under drought stress enhanced maize seedling growth parameters, such as plant biomass (approximately 43%), chlorophyll concentration (approximately 14%), and antioxidant enzyme capacity. Besides, considerable changes were observed in the rhizosphere microbial community’s composition and structure. Recent studies have highlighted the positive effects of MF on drought tolerance in various plant species, including medicinal crops like basil. For example, Zare [18] evaluated basil plants inoculated with *Glomus hoi*, exposed to 60% and 90% of FC. They found that MF accumulation enhanced plant growth parameters, phosphorous content, and antioxidant activities. Similarly, in MF-inoculated *Pelargonium graveolens*, increases in nutrient concentration, plant biomass, and essential oil content were observed [19].

Although numerous studies have investigated the individual effects of SA and MF on various plant species, no research has yet evaluated their combined impact on marjoram. This study aims to address this gap by exploring the interaction between SA and MF symbiosis and examining their collective influence on drought tolerance in marjoram. This research specifically hypothesizes that integrating SA and MF will synergistically enhance marjoram’s tolerance to drought by improving its morphological, physiological, and biochemical traits. Specifically, the combined application is expected to regulate ROS levels, boost antioxidant enzyme activity, and improve photosynthetic efficiency under drought conditions. The study primarily focused on understanding the interaction between SA and MF symbiosis and their combined impact on drought tolerance. Accordingly, the study addresses the following research question: Can the combined application of SA and MF improve marjoram’s drought tolerance by enhancing its morphological, physiological, and biochemical traits? The findings of this research have practical implications at both small and large scales. For small-scale applications, the results can guide herb farmers in improving marjoram’s tolerance to drought stress. At a larger scale, the findings can inform policymakers in incorporating these adaptive strategies into policies aimed at mitigating the impacts of drought stress on agricultural systems.

## Materials and methods

### Study area and experimental design

**Fig. 1:**
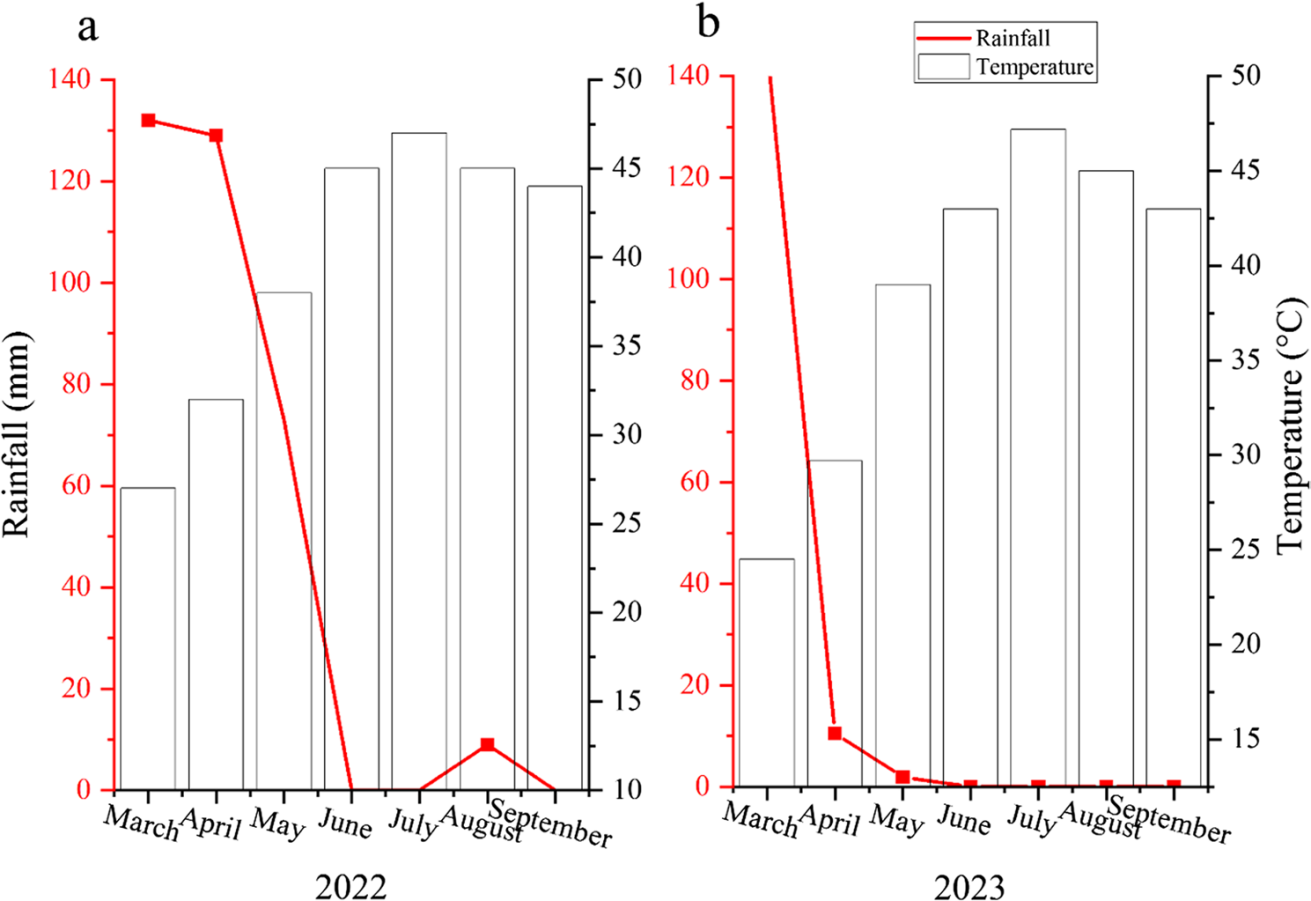
Rainfall and temperature conditions over two years of (**a**) 2022, and (**b**) 2023 in the study area

This research was performed in an experimental field located in Fars Province, Iran (51°39’15” E and 29°27’ N, with 950 m above sea level) between 2022 and 2023. The climatic parameters of the study area are depicted in Fig. 1. The study was implemented as a split factorial within a randomized complete block design with three replications. Accordingly, drought stress (D) was the primary factor at three levels (90% (D0), 70% (D1), and 35% (D2) field capacity (FC)), and the secondary factor included two sub-factors: mycorrhizal fungi (non-inoculated (M0) and inoculated with *Glomus hoi* (M1)) and different concentrations of salicylic acid (0 mg L^−1^ (SA0), 100 mg L^−1^ (SA100), and 300 (SA300) mg L^−1^). SA concentration was chosen based on our preliminary trials. The experimental field soil was loamy, with a depth of 60 to 90 cm. Table 1 presents some of the physiochemical properties of the field soil. Based on the soil physiochemical analysis, no fertilization was performed. The prepared plots were divided into three equal blocks following a partitioning method, and the experimental treatments were applied. Each block comprised 18 plots, with each plot containing three planting rows, each 5 m long, spaced 50 cm apart, and with 30 cm spacing between plants within the rows. The blocks were separated by a distance of 2 m.

**Table 1:** Interaction effects of drought stress (D) and Salicylic acid (SA) on plants morphologic traits

Treatments		Lateral branch		EL (%)		RWC (%)	
		M0	M1	M0	M1	M0	M1
D0	SA0	37.82 ± 2.5 f	42.4 ± 2.3 ed	24.01 ± 1.5 ef	20.56 ± 2.3ef	75.94 ± 2.5 d	87.40 ± 2.4 cb
	SA100	46.33 ± 3.2 cb	50.81 ± 2.3a	24.07 ± 2.1 e	18.79 ± 2.2ef	79.99 ± 2.8 cd	95.12 ± 2.8 a
	SA300	50.47 ± 2.3 a	49.25 ± 2.2 ab	23.29 ± 1.2ef	18.52 ± 2.4f	87.13 ± 2.2 cb	97.57 ± 2.1 a
D1	SA0	37.5 ± 2.7f	38.18 ± 2.4f	50.6 ± 1.3b	40.48 ± 2.3c	77.04 ± 2.3d	80.64 ± 2.3 cd
	SA100	39.25 ± 3.2 ef	39.91 ± 2.3 ef	40.45 ± 2.5c	40.28 ± 2.2c	86.23 ± 2.2cb	91.69 ± 2.2 ab
	SA300	43.63 ± 2.2 cd	43.71 ± 2.3 cd	40.35 ± 1.3c	40.15 ± 2.3c	92.67 ± 2.6ab	93.09 ± 2.1 ab
D2	SA0	25.55 ± 2.1 g	24.18 ± 2.1 g	63.02 ± 2.1a	43.05 ± 2.1c	36.415 ± 2.0 h	55.80 ± 0.8 g
	SA100	25.92 ± 2.2 g	38.19 ± 2.2f	43.28 ± 1.3c	43.28 ± 3.2c	68.24 ± 1.9 ef	74.68 ± 1.1 ed
	SA300	39.81 ± 2.1 ef	40.73 ± 2.1 edf	43.49 ± 2.1c	33.13 ± 3.0d	65.33 ± 1.5 f	73.09 ± 1.2 ed

### Treatments application

The used marjoram seeds variety *Yazdi* was obtained from the Research Institute of Forests and Rangelands of Iran. Furthermore, the mycorrhizal fungus, *Glomus hoi*, was sourced from the Organic Plant Protection Clinic (Arbuscular Mycorrhizal Biological Fertilizer in Iran) located in Hamadan. Seeds were sown in seedling trays in a greenhouse at a temperature of 20 ± 5 °C in March of both years. After three weeks and complete germination, the seedling trays were exposed to open air for one week. In April of both years, the plants, at the 4–6 leaf stage, were transplanted to the main field along with the potting soil. Before planting, the field soil was inoculated with mycorrhizal fungi at a rate of 80 kg ha^−1^, distributed at a depth of 5–10 cm along each planting row. The inoculum was manually applied to ensure uniform distribution along each planting row, thereby optimizing root-fungal contact. It was carefully incorporated into the soil at the specified depth using hand tools and immediately irrigated to promote spore germination and successful fungal establishment. The mycorrhizal inoculum used in this study contained a mixture of *Glomus* spores and fungal hyphae. The inoculum was prepared to include both spores and mycelium to ensure successful establishment and colonization in the rhizosphere. The exact spore density of the inoculum was approximately 200 spores per gram, providing a sufficient quantity of propagules to support effective symbiosis. Besides, the average soil moisture content at the planting stage was 26.21%. To accurately determine soil moisture status, tensiometers and gypsum blocks were simultaneously used in the experimental plots [20]. A pressure plate device was employed to obtain the FC and permanent wilting point (PWP). The volumetric soil moisture at FC was 32.4%, and at the PWP, it was 15.2%. The following equation was used to determine the water requirement for each irrigation regime [20].

1 $$\:Dn=\:\frac{FC-pwp}{100}\:\times\:pb\times\:Dr\times\:F$$

Where in this equation, (D_n_) represents the amount of water per irrigation regime (mm), (FC) is the gravimetric soil moisture content at FC, PWP is the gravimetric soil moisture content at the permanent wilting point, (ρb) is the soil bulk density (1.45 g cm³), (Dr) is the effective root depth (mm), and (F) is the soil moisture depletion coefficient (%). Water was transferred from the source to the experimental plots using polyethylene pipes and a pump. Irrigation was carried out immediately after planting, and tensiometers were installed at a depth of 35 cm in the experimental plots for all treatments. To ensure the establishment of the seedlings, no drought stress was applied for the first 15 days. After this initial period, the drought stress treatments were applied as described. Accordingly, drought stress began five days after the first SA foliar application on November 17 and continued until the harvest on March 10, Lasting a total of 114 days after sowing (DAS).

The SA foliar spray treatment was first applied 10 days after transplanting the seedlings to the field to ensure their establishment. It was subsequently repeated at 25-day intervals for the second and third applications [21]. It is noteworthy that during the growth stage, no rainfall occurred in the region. Weed control was performed mechanically by hand in three stages.

### Measurements

#### Morphologic parameters

At the full flowering stage, ten plants from each treatment were harvested at soil level, packaged, and transported to the Laboratory in a cool, ice-filled container. Plant Height was measured from the soil surface to the tip of the tallest inflorescence, and then the fresh weight of the plants was recorded. The harvested tissues were dried at 40 °C for 72 h, and plant dry weight was measured.

To determine electrolyte leakage (EL), leaf samples were placed in test tubes containing 8 mL of distilled water and kept at room temperature for 24 h. The initial EL (EL_1_) was measured, after which the samples were placed in a 100 °C bath for 20 min to release all electrolytes, and then cooled to 25 °C to determine the final EL (EL_2_). The EL value was calculated using Eq. 2 [22]:

2 $$\text{EL}(\%)=\frac{EL1}{EL2}\:\times\:100$$

Relative water content (RWC) was determined by preparing fresh leaf slices (2 cm² diameter). The slices were immediately weighed (Fresh mass) and then floated on deionized distilled water for 24 h in a dark environment to achieve saturation. The excess water on the slices was removed, and the turgor mass was measured. The disc’s dry mass was measured following a 48-hour drying period at 40 °C (Dry mass). RWC was calculated using Eq. 3 [23].

3 $$\text{RWC}=\frac{Fresh\:mass-dry\:mass}{Turgor\:mass-dry\:mass}\:\times\:100$$

#### Photosynthetic measurements

The amounts of chlorophyll a, b, total chlorophyll, and carotenoids in the leaves were measured using the dimethyl sulfoxide (DMSO) method. Initially, 0.2 g of fresh leaf tissue was placed in a test tube, followed by the addition of 10 mL of DMSO. The test tubes were then kept in a dark place in the Laboratory for 24 h. Then, the extract was filtered, and the leaf tissues were discarded. Subsequently, 200 µL from each sample were placed into a microplate, and the absorbance of the extracts at wavelengths of 645 nm, 663 nm, and 470 nm was read using a spectrophotometer (Epoch Microplate Spectrophotometer, BioTek Instruments, USA). Notably, DMSO was used as the blank. The concentrations of chlorophyll a, b, total chlorophyll, and carotenoids in the samples were reported in mg g^−1^ of fresh leaf weight and calculated using the formulas introduced by [24].

4 $$\text{Chlorophyll a}\,(\text{mg}\,\text{g}^{-1}\text{FW})=\frac{\text{E}\text{x}\text{t}\text{r}\text{a}\text{c}\text{t}\:\text{v}\text{o}\text{l}\text{u}\text{m}\text{e}\:\left(12.7\:\times\:\text{A}663\right)-(2.69\:\times\:\text{A}\:645)}{\text{S}\text{a}\text{m}\text{p}\text{l}\text{e}\:\text{w}\text{e}\text{i}\text{g}\text{h}\text{t}}$$

5 $$\text{Chlorophyll a}\,(\text{mg g}^{-1}\,\text{FW})=\frac{\text{E}\text{x}\text{t}\text{r}\text{a}\text{c}\text{t}\:\text{v}\text{o}\text{l}\text{u}\text{m}\text{e}\:\left(22.9\:\times\:\text{A}645\right)-(4.68\:\times\:\text{A}\:663)}{\text{S}\text{a}\text{m}\text{p}\text{l}\text{e}\:\text{w}\text{e}\text{i}\text{g}\text{h}\text{t}}$$

6 $$\text{Total chlorophyll}\,(\text{mg g}^{-1}\,\text{FW})=\frac{\text{E}\text{x}\text{t}\text{r}\text{a}\text{c}\text{t}\:\text{v}\text{o}\text{l}\text{u}\text{m}\text{e}\:\left(20.2\:\times\:\text{A}645\right)+(8.02\:\times\:\text{A}\:663)}{\text{S}\text{a}\text{m}\text{p}\text{l}\text{e}\:\text{w}\text{e}\text{i}\text{g}\text{h}\text{t}}$$

7 $$\text{Carotenoid}\, (\text{mg g}^{-1}\,\text{FW})=\frac{(\left(1000\times\:\text{A}470\right)-(1.82\:\text{C}\text{h}\text{l}\text{a}-85.02\:\text{C}\text{h}\text{l}\:\text{b})\:}{\text{S}\text{a}\text{m}\text{p}\text{l}\text{e}\:\text{w}\text{e}\text{i}\text{g}\text{h}\text{t}}$$

#### Physiochemical measurements

To measure proline content, 0.1 g of dry leaf powder was weighed and placed into a test tube, to which 10 mL of methanol was added. The test tubes were then placed in a water bath (in complete darkness) at 40 °C for three hours. The homogenized supernatant was then filtered using Whatman filter paper. Proline concentration in the samples was measured using a 1% (w/v) ninhydrin reagent solution, prepared by mixing 60% acetic acid, 20% methanol, 1 g ninhydrin, and distilled water. Next, 100 µL of the extract and 200 µL of the ninhydrin reagent were transferred into microtubes, which were then incubated at 95 °C for 20 min. The samples were immediately cooled and then centrifuged at 2500 rpm (559 xg) for one minute. Finally, 200 µL of each sample was transferred to microplate wells and read at 520 nm using an Epoch Microplate Spectrophotometer (BioTek Instruments, USA). Methanol and the reaction mixture were used as blanks. Proline content was calculated based on a standard curve of pure L-proline and reported as mg g^−1^ of dry weight [25].

Besides, to determine soluble sugar content, 0.1 g of dry powdered leaf tissue was weighed and placed in a falcon tube, to which 10 mL of 80% ethanol was added. The samples were centrifuged at 5000 rpm (2.23 xg) for 10 min. The supernatant was transferred to a new falcon tube, and another 10 mL of 80% ethanol was added to the remaining pellet. The samples were centrifuged again at 5000 rpm (2.23 xg) for 10 min, and the supernatant was combined with the previous one. A 5% phenol solution was prepared, and 25 µL of the target solution was placed into microplate wells. Subsequently, 25 µL of the 5% phenol solution was added to each well containing the extract. Immediately, 125 µL of concentrated sulfuric acid was added to each well. The samples were then incubated at 25–30 °C for 30 min. Absorbance was measured at 490 nm using an Epoch Microplate Spectrophotometer (BioTek Instruments, USA). A glucose solution, in varying concentrations, was used to create a standard curve, and the soluble carbohydrate content was reported as mg g^−1^ of dry weight [26].

#### Antioxidant measurements

##### Extraction for measurement of antioxidant enzymes

To extract antioxidant enzymes, a fresh leaf sample (0.5 g) was thoroughly ground and powdered in a mortar with Liquid nitrogen. Subsequently, 2 mL of cold 50 mM potassium phosphate buffer (pH 7.0), containing 2 mM ethylenediaminetetraacetic acid (EDTA) and 1% (w/v) polyvinylpyrrolidone (PVP), was added to the powdered sample and homogenized well. The resulting homogenate was centrifuged at 13,000 rpm (15.115 xg ) for 10 minutes at 4°C using a refrigerated centrifuge. The supernatant was collected as the desired extract and stored at -80°C until further enzyme activity assays [27].

To measure the activity of SOD, 50 µL of enzyme extract was added to 1 mL of reaction mixture containing 50 mM potassium phosphate buffer (pH 7.8), 13 mM L-methionine, 75 µM nitroblue tetrazolium (NBT), 4 µM riboflavin, and 0.1 mM EDTA. The riboflavin solution was prepared separately in darkness and added to the reaction mixture as the final component. The cuvettes were then placed under a light source (two 15 W fluorescent lamps) for 15 min. The reaction was stopped by turning off the Lights and placing the samples in darkness. The absorbance of each sample was read at 560 nm using a spectrophotometer (Model 7315, JENWAY, UK) [27].

Besides, the POD activity was measured using the method described by [28]. This method is based on the increase in absorbance due to the oxidation of guaiacol in the presence of hydrogen peroxide at a wavelength of 470 nm, measured with a spectrophotometer over 2 min at 10-second intervals. The reaction mixture consisted of 2 mL containing 100 µL of enzyme extract, 700 µL of 50 mM potassium phosphate buffer (pH 7.0), and 100 µL of 20 mM guaiacol. The reaction was initiated by adding 100 µL of 40 mM hydrogen peroxide. The enzyme activity was calculated using an extinction coefficient of 26.6 mM^−1^ cm^−1^.

Moreover, CAT activity was assessed using the method by [28]. Accordingly, the enzyme activity was determined by measuring the decrease in absorbance due to the decomposition of hydrogen peroxide (H₂O₂) over one minute using a spectrophotometer (Model 7315, JENWAY, UK) at 240 nm. The reaction mixture consisted of 3 mL, including 2800 µL of 50 mM potassium phosphate buffer (pH 7.0), 100 µL of 0.1 M hydrogen peroxide, and 100 µL of enzyme extract. The reaction was initiated by adding the enzyme extract to the reaction mixture. The enzyme activity was calculated using the extinction coefficient of 39.4 mM^−1^ cm^−1^.

### Data analysis

Data were analyzed using SAS 9.1 software, and mean comparisons were performed based on Duncan’s multiple range test. Graphs were generated using Origin Pro 2021 software.

## Results

### Morphologic parameters

**Fig. 2:**
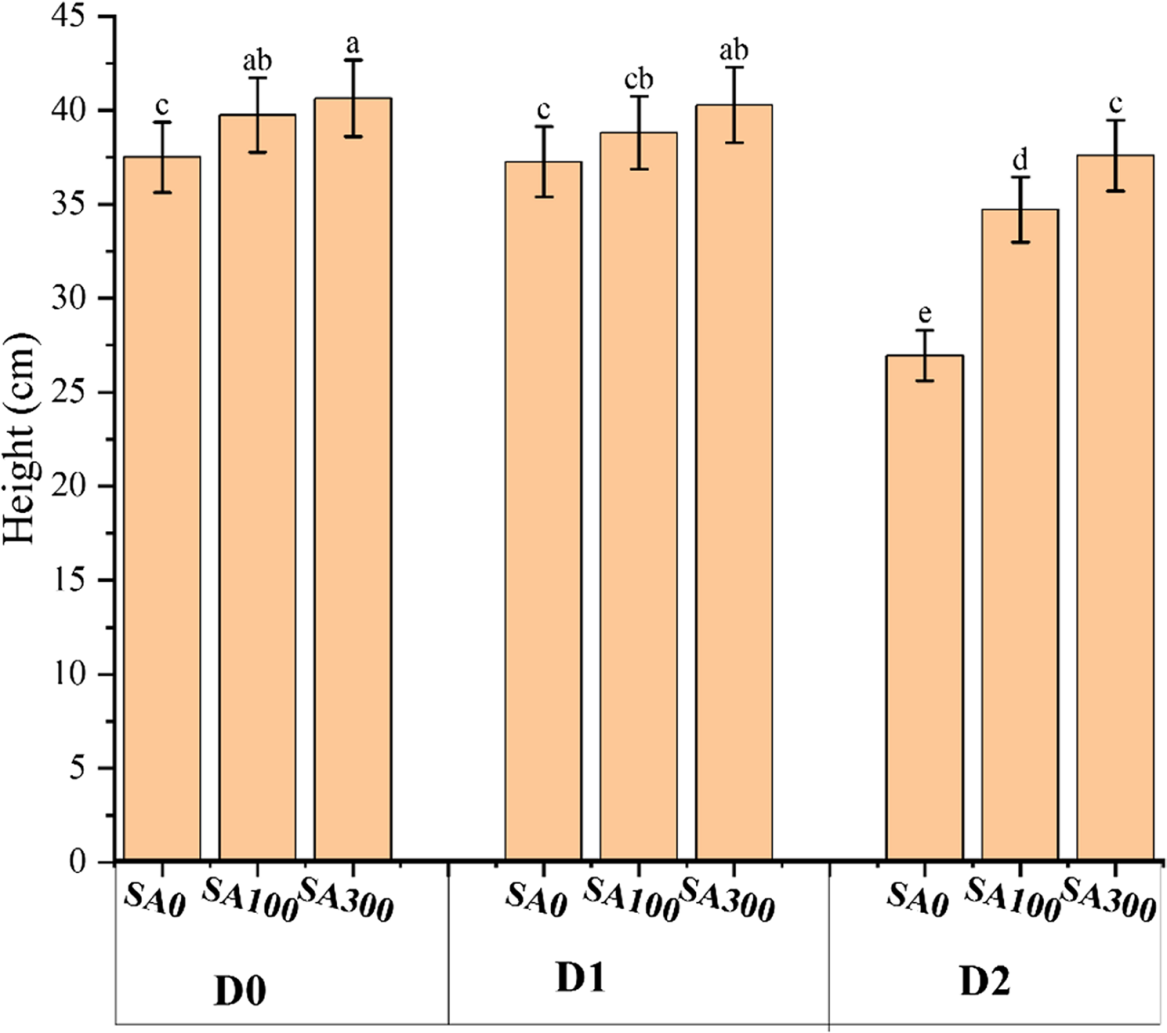
Interaction effects of drought stress (D) and salicylic acid (SA) on Marjoram plants height. D0: no drought stress or 90% of field capacity (FC); D1: drought stresses at 70% of FC; D2: drought stress at 35% of FC; SA0: no salicylic acid; SA100: salicylic acid treatment at 100 mg L^−1^; and SA300: salicylic acid treatment at 300 mg L^−1^. Data represent the mean of three replicates ± SE. Means with different letters have a significant difference (*P* ≥ 0.01)

The analysis of variance (ANOVA) results (Table S2, Supplementary file) revealed that both the main and interaction effects of year, drought stress, salicylic acid, and mycorrhizal fungi significantly influenced the studied parameters. Accordingly, Fig. 2 exhibited that drought stress decreased marjoram height under D2 (severe stress conditions) by an average of 15% compared to D0 (control). However, this reduction was compensated by the SA application. Specifically, under D2 conditions, SA300 increased plant Height by 29% compared to SA0 in the same treatment. Furthermore, the number of Lateral branches decreased as drought stress severity increased, with a reduction of approximately 11% at D1 and 51% at D2. However, the application of SA significantly enhanced this parameter at a concentration of 300 mg L^−1^. Under D0 conditions, SA300 increased the number of Lateral branches by 9% compared to SA100, and by 13% compared to SA0. A similar trend was observed under D2 stress levels. Notably, mycorrhizal fungi did not have a significant effect under various water deficit conditions. As for the EL parameter, higher drought stress conditions significantly increased EL, whereas SA and fungi application mitigated this damage. Under D2 conditions with no SA application, EL increased by 61% compared to D0, but this increase was mitigated by 31% under the same stress conditions (D2) with fungi application (M1) (Table 1). Conversely, RWC decreased under higher drought stress conditions. However, SA application significantly mitigated the reduction in RWC under both drought conditions, increasing it by 13% in D1 and 44% in D2 (Table 1). Fungi application also significantly influenced RWC, particularly under severe stress conditions. Accordingly, M1 treatment (mycorrhizal fungi application) enhanced RWC by 10% and 8% under D2-SA300 and D2-SA100 treatments, respectively (Table 1).

### Photosynthetic parameters

**Fig. 3:**
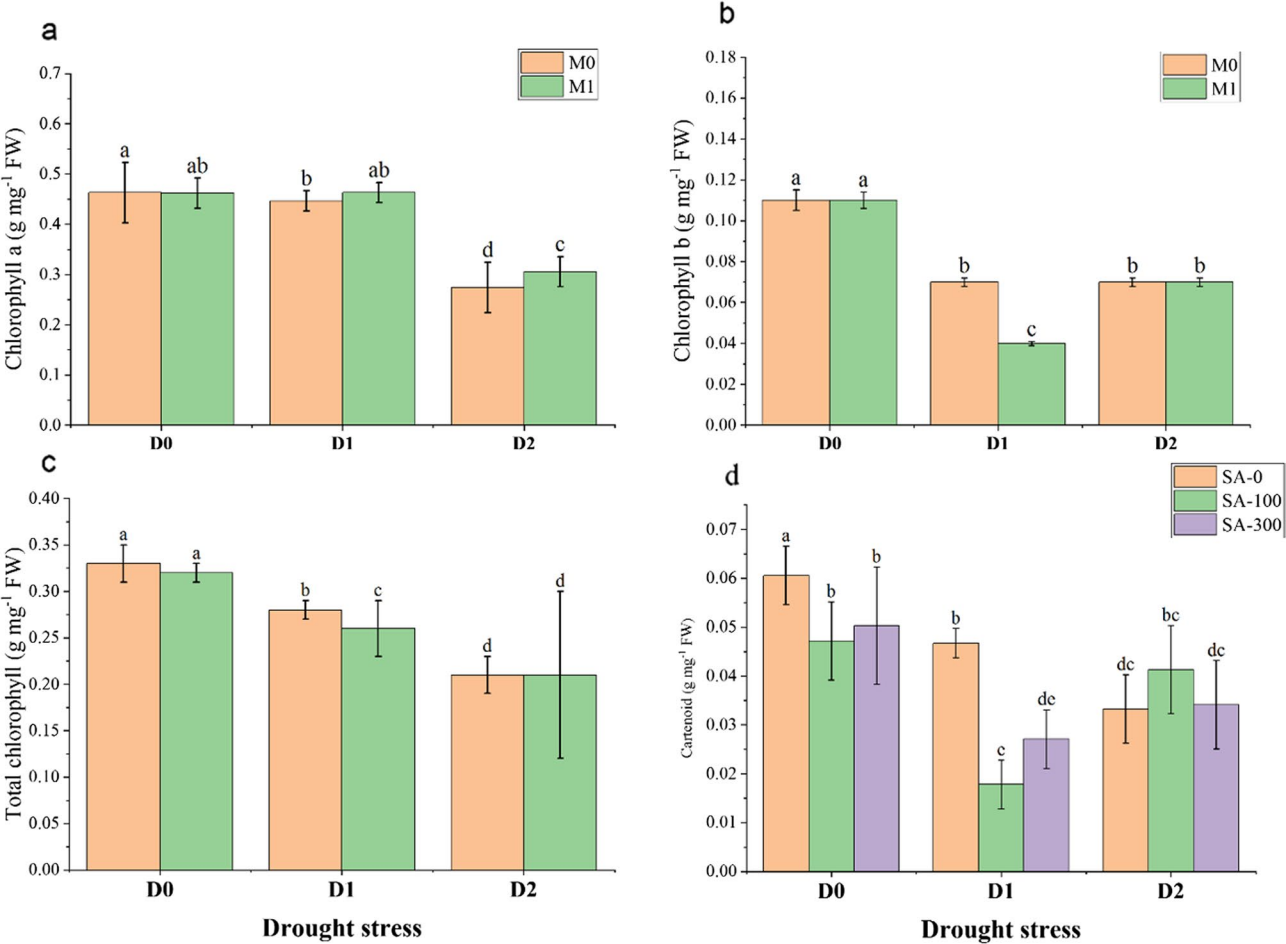
Interaction effects of drought stress (D) and salicylic acid (SA) on Marjoram plants (**a**) chlorophyll a content; (**b**) chlorophyll b content; (**c**) total chlorophyll content; (**d**) carotenoid content. D0: no drought stress or 90% of field capacity (FC); D1: drought stresses at 70% of FC; D2: drought stress at 35% of FC; SA0: no salicylic acid; SA100: salicylic acid treatment at 100 mg L^−1^; and SA300: salicylic acid treatment at 300 mg L^−1^. M0: No fungi inoculation; M1: with fungi inoculation. Data represent the mean of three replicates ± SE. Means with different letters have a significant difference (*P* ≥ 0.01)

Regarding chlorophyll pigments, ANOVA results exhibited that the main effects of year, drought stress, SA concentrations, and mycorrhizal fungi, along with their interactions, significantly affected these parameters (Table S3, Supplementary file). Figure 3 indicated that drought stress significantly decreased chlorophyll levels, with a more pronounced effect at the D2 level. No significant difference was observed in chlorophyll a content between D0 and D1, while this value decreased significantly by nearly 60% at D2 (Fig. 3a). Notably, this reduction was mitigated by 10% with fungi application (M1). Conversely, chlorophyll b content was significantly reduced by 36% under D1, while no significant difference was observed between D1 and D2. Furthermore, M1 did not significantly affect this parameter (Fig. 3b). As for total chlorophyll content, a decreasing trend was observed with increasing drought severity (Fig. 3c). The lowest value was recorded at D2 (0.21 g mg^−1^ FW), which was not affected by fungi application. On the other hand, carotenoid content was impacted by drought stress (Fig. 3d), with a decrease under more severe drought conditions. Notably, the SA application did not enhance carotenoid content under D0 and D1 compared to SA0 in the same treatments. However, at D2, SA100 increased carotenoid content by 19% compared to SA0.

### Physiochemical traits

**Fig. 4:**
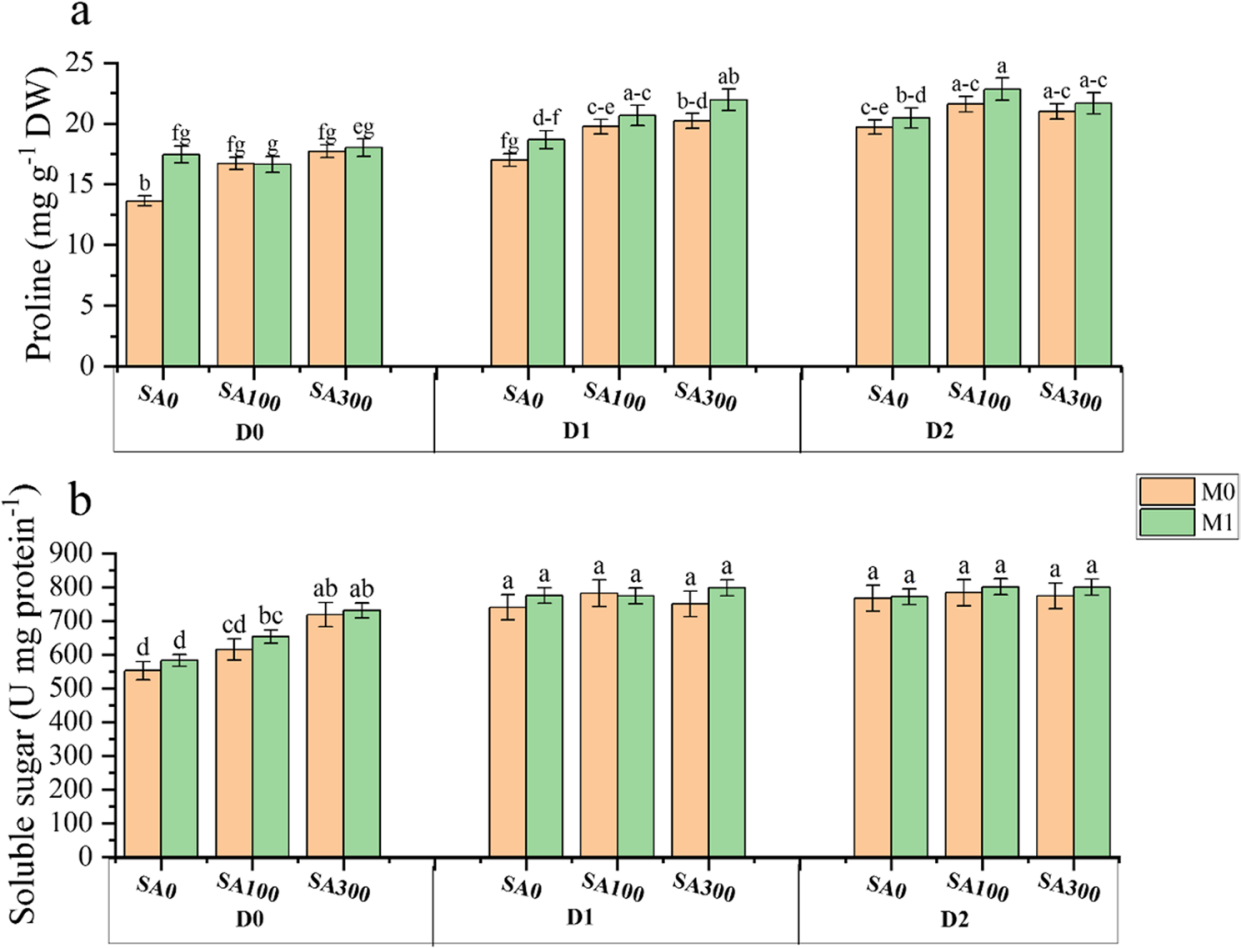
Interaction effects of drought stress (D), salicylic acid (SA), and mycorrhiza fungi (M) on Marjoram plants (**a**) proline content; (**b**) soluble sugar. D0: no drought stress or 90% of field capacity (FC); D1: drought stress at 70% of FC; D2: drought stresses at 35% of FC; SA0: no salicylic acid; SA100: salicylic acid treatment at 100 mg L^−1^; and SA300: salicylic acid treatment at 300 mg L^−1^. M0: No fungi inoculation; M1: with fungi inoculation. Data represent the mean of three replicates ± SE. Means with different letters have a significant difference (*P* ≥ 0.01)

ANOVA results exhibited that the main effects of year, drought stress, SA concentrations, and mycorrhizal fungi, along with their interactions, significantly impacted proline and soluble sugar content (Table S3, Supplementary file). The combined interaction of drought stress, SA concentration, and mycorrhizal fungi significantly influenced proline content at the 1% probability level (Fig. 4). Proline content increased under D1 and D2 stress conditions while irrigating marjoram plants at 90% field capacity (D0) decreased this parameter to approximately17.63 mg g^−1^ DW. The SA100 and SA300 treatments increased proline content by approximately 10% across all treatments. Mycorrhizal fungi inoculation further increased proline content in leaf tissue (Fig. 4a). Under D1 and D2 stress conditions, SA100 and SA300 foliar application combined with mycorrhizal inoculation(M) significantly increased proline content in plant leaf tissue by approximately 12%. A similar trend was observed for soluble sugar content. Accordingly, irrigating plants at D1 and D2 treatments increased soluble sugar content and remained stable, while the D0 stress condition enhanced this parameter. Besides, the SA100 and SA300 treatments increased soluble sugar content by approximately 6% (Fig. 4b).

### Antioxidant enzymes parameters

**Fig. 5:**
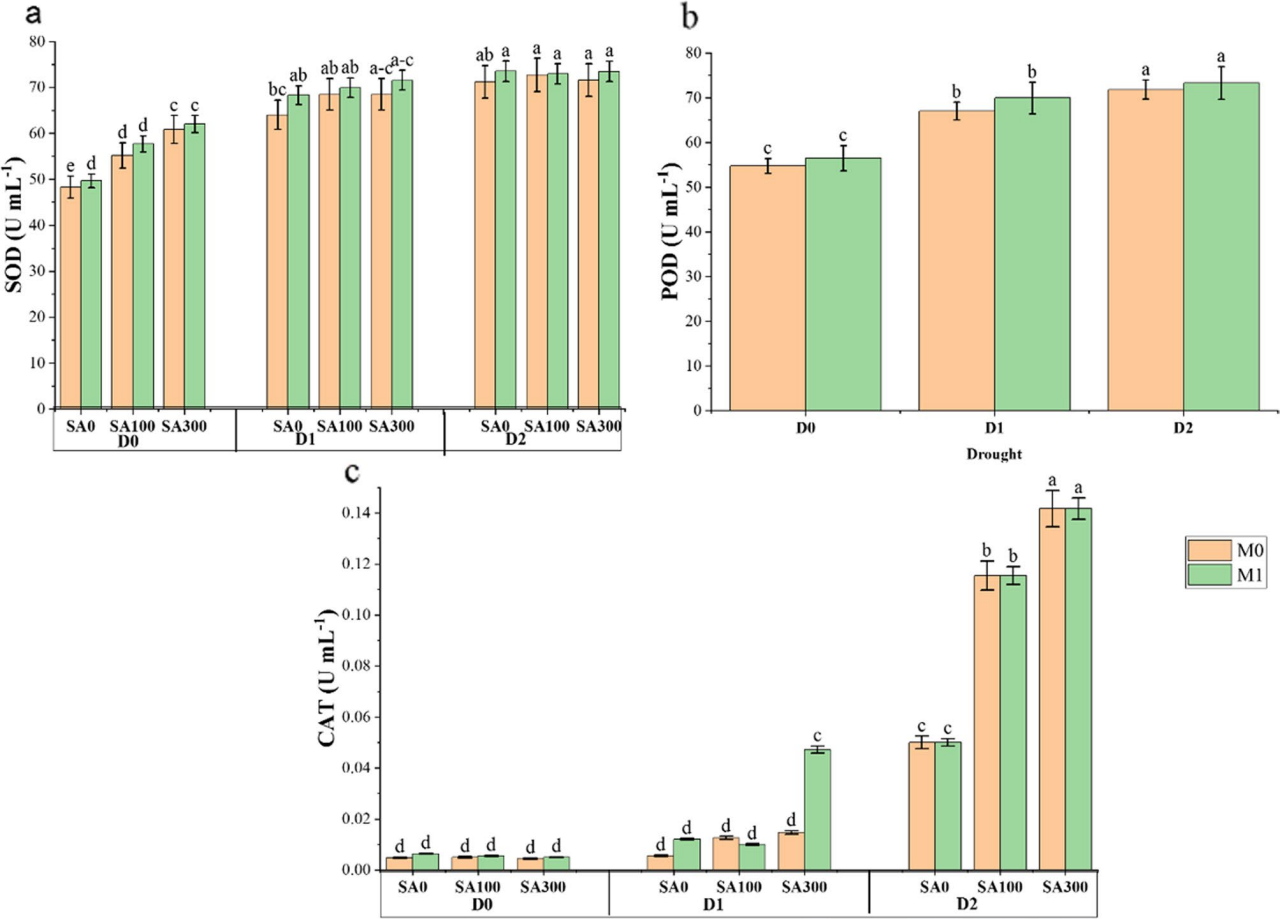
Interaction effects of drought stress (D) and salicylic acid (SA) on Marjoram plants (**a**) SOD: superoxide dismutase content; (**b**) D and mycorrhiza fungi (M) on POD: peroxidase content; (**c**) CAT: catalase content. D0: no drought stress or 90% of field capacity (FC); D1: drought stresses at 70% of FC; D2: drought stresses at 35% of FC; SA0: no salicylic acid; SA100: salicylic acid treatment at 100 mg L^−1^; and SA300: salicylic acid treatment at 300 mg L^−1^. M0: No fungi inoculation; M1: with fungi inoculation. Data represent the mean of three replicates ± SE. Means with different letters have a significant difference (*P* ≥ 0.01)

Enzymatic activities increased with drought stress severity. ANOVA results showed that the main effects of year, drought stress, SA concentrations, and mycorrhizal fungi, along with their interactions, significantly influenced all enzyme activities at the 1% probability level (Table S3, Supplementary file). Plants under D1 and D2 stress had the highest SOD activity (approximately 70 U mL^−1^), whereas those under normal conditions (D0) had significantly lower activity (60 mL^−1^). Foliar application of SA300 and mycorrhizal fungi inoculation (M1) both significantly increased SOD activity. Interaction analysis indicated that plants under D1 and D2 drought stress, treated with SA100 or 300 (with or without mycorrhizal fungi), showed the highest SOD activity (Fig. 5a). Similarly, POD activity increased under drought stress, showing a 27% increase at D2 compared to D0 (Fig. 5b), with fungi application also enhancing POD content. Besides, CAT activity was highest in plants subjected to D2 stress (approximately 75 U mg^−1^), while those under normal (D0) and medium drought stress (D1) conditions showed significantly lower activity. Accordingly, CAT content increased with drought severity, rising from 0.01 U mL^−1^ at D0 to an average of 0.12 U mL^−1^, a 91% increase (Fig. 5c). Further fluctuations were observed in D2 treatments, where SA significantly increased CAT content from 0.06 U mL^−1^ in the control (SA0) to approximately 0.12 U mL^−1^ at SA100 and 0.14 U mL^−1^ at SA300. Although fungi application did not have a significant effect, it still contributed to an increase in enzyme activity (Fig. 5c).

### Factor analysis and correlation results

**Fig. 6:**
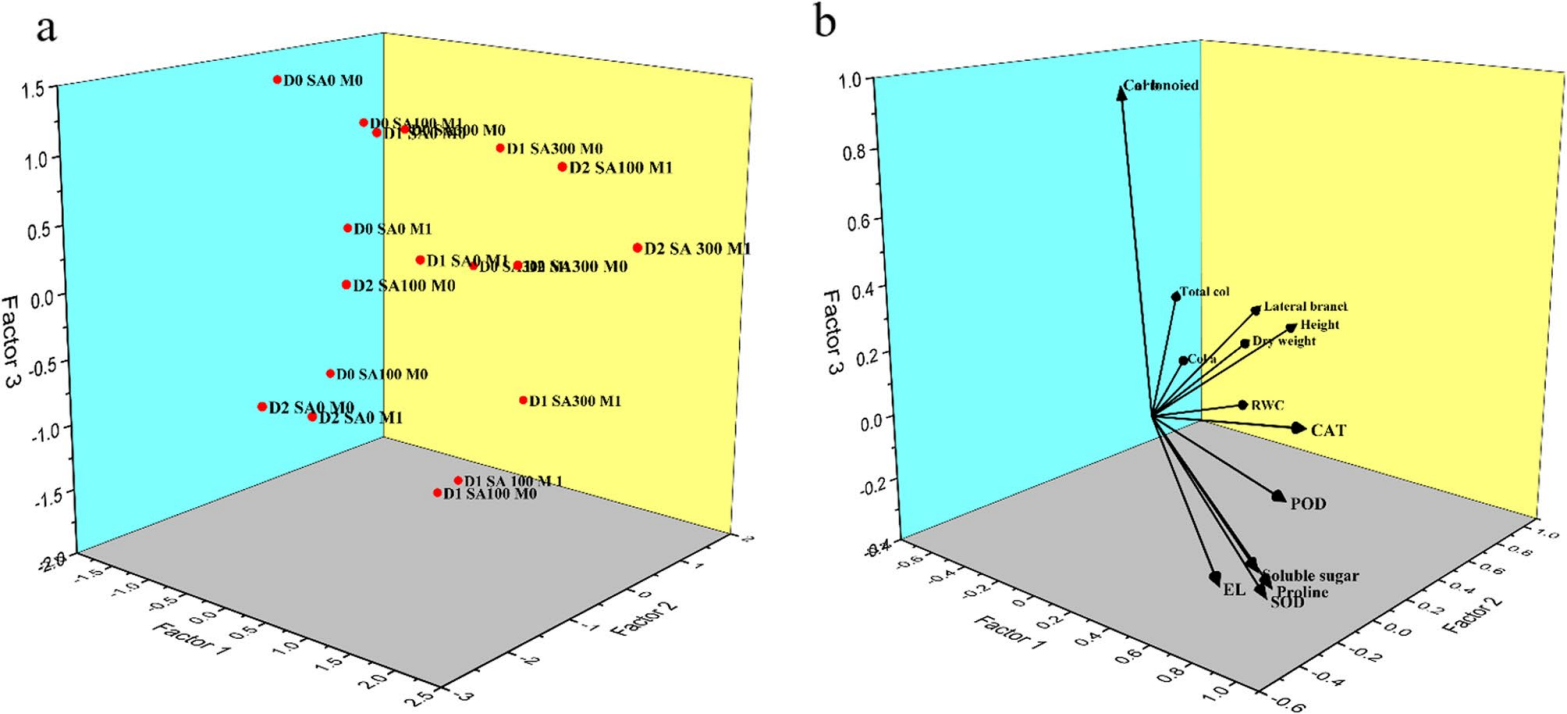
Factor analysis of (**a**) treatments on (**b**) studied parameters. D0: no drought stress or 90% of field capacity (FC); D1: drought stresses at 70% of FC; D2: drought stress at 35% of FC; SA0: no salicylic acid; SA100: salicylic acid treatment at 100 mg L^−1^; and SA300: salicylic acid treatment at 300 mg L^−1^. M0: No fungi inoculation; M1: with fungi inoculation. Col a: Chlorophyll a; Col b: Chlorophyll b; Total col: Total chlorophyll; EL: Electrolyte leakage; POD: Peroxidase; CAT: Catalase; SOD: Superoxide dismutase

**Fig. 7:**
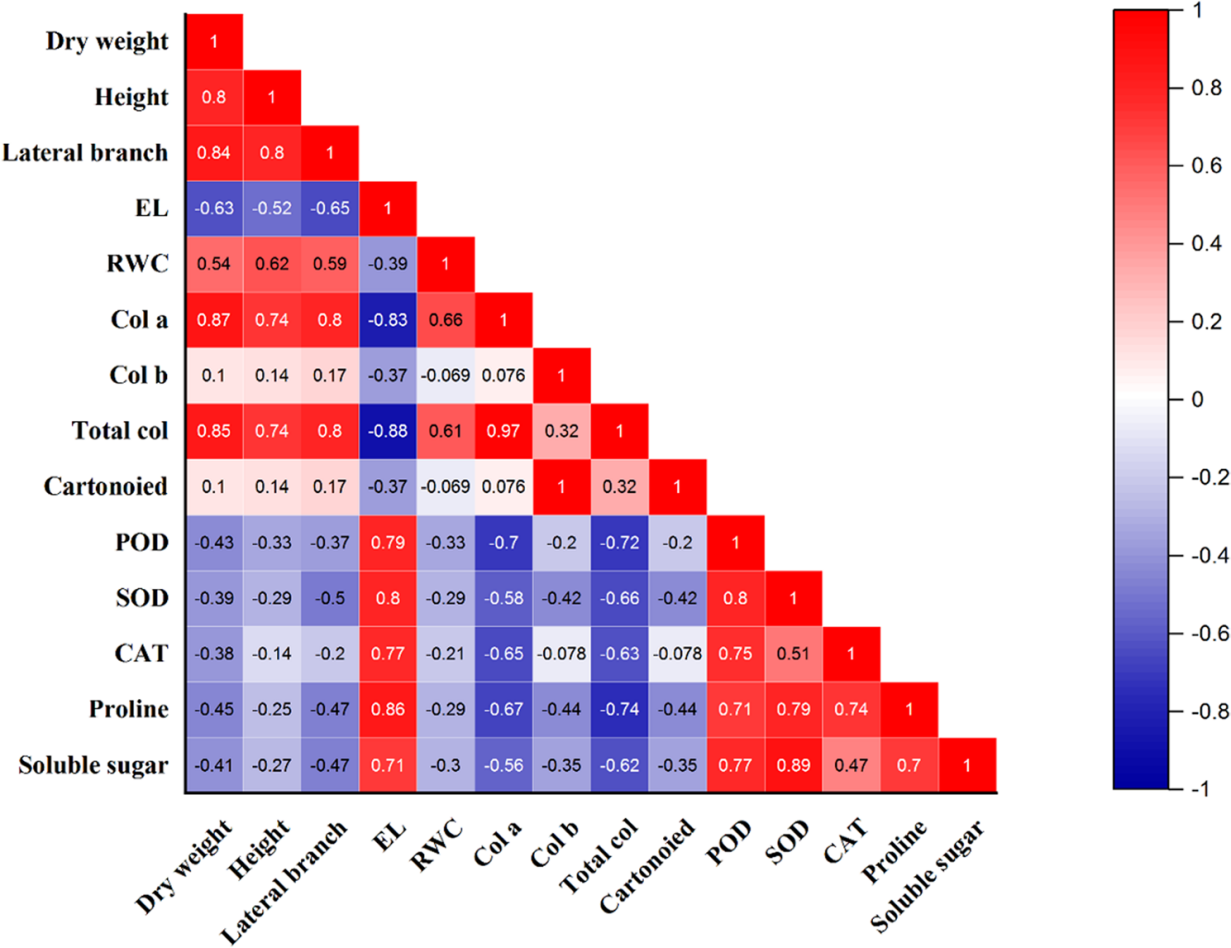
Pearson correlation between plant dry weight and various physiological traits. Col a: Chlorophyll a; Col b: Chlorophyll b; Total col: Total chlorophyll; EL: Electrolyte leakage; POD: Peroxidase; CAT: Catalase; SOD: Superoxide dismutase

Furthermore, factor analysis results (Fig. 6) exhibited that factor 1 (D1 SA100 M0, D1 SA100 M1) predominantly explained the variance in the variables (56.60%), encompassing two enzymes such as POD and SOD, EL, proline, and soluble sugars. Conversely, Factor 2 (including D2, SA, and fungi treatments) accounted for 2.41% of the variance, primarily associated with morphological traits (height, lateral branch, and dry weight), RWC, and CAT (Fig. 6b). On the other hand, Factor 3 (mainly related with D0 condition), explaining the least variance (1.63) was associated with photosynthetic parameters (Fig. 6b). Besides, a positive and significant correlation was found between plant dry weight and morphological traits (including height (*r* = 0.8), lateral branch (*r* = 0.84), RWC (*r* = 0.54), chlorophyll a (*r* = 0.87) and total chlorophyll (*r* = 0.85). Whereas, there was a negative and significant correlation between plant dry weight and EL (*r*=−0.63), POD (*r*=−0.43), SOD (*r*=−0.39), CAT (*r*=−0.38), proline (*r*=−0.45), and soluble sugar (*r*=−0.41) (Fig. 7).

## Discussion

This study investigated the mitigated effects of SA and mychorizal fungi on morphological, physiological, and biochemical parameters of Marjoram plants exposed to mild (70% of FC) and severe (35% of FC) drought stress conditions over two years of 2023 and 2024. Results revealed that the studied treatments significantly affected the mentioned parameters. Accordingly, a significant reduction in plant height under mild (D1) and severe drought (D2) conditions compared to the control was observed (Fig. 2). Drought typically inhibits cell expansion due to water deficit, leading to reduced plant growth [29]. However, the SA foliar application mitigated this reduction. SA is known to act as a signaling molecule that modulates plant stress responses [7]. It helps maintain water use efficiency and can activate antioxidant defense mechanisms, thus promoting better growth under stressful conditions [7]. The enhanced number of lateral branches with SA application (300 mg L^−1^) further supports the role of SA in mitigating drought effects on plant morphology. On the other hand, the sharp increase in EL under D2 treatment reflects the extent of membrane damage caused by water stress (Table 1). High EL indicates that cellular membranes are losing their integrity, often a result of oxidative damage during stress [4]. The finding that SA and mycorrhizal fungi application reduced EL is aligned with previous studies suggesting that both SA and mycorrhizae help stabilize cell membranes [4, 24]. SA’s contribution to strengthening antioxidant systems, coupled with mycorrhizal fungi’s ability to improve water uptake and nutrient absorption, likely contributed to the observed reduction in EL. Furthermore, the decline in RWC under drought conditions is a typical indicator of water deficit within plant tissues. However, the application of SA and mycorrhizal fungi significantly mitigated this decline, which is crucial for maintaining physiological processes and plant survival during drought. Increased RWC under SA treatment (Table 1), especially under severe drought, indicates that SA helps in maintaining water balance by enhancing root development or reducing transpiration rates, which aligns with recent findings [4, 5]. Mycorrhizal fungi’s contribution to improving RWC is also well-documented [13], as they can extend the root surface area and help plants access water from the soil more effectively. Besides, our results indicated that photosynthetic parameters decreased under drought stress conditions (Fig. 3). Similarly, Emrahi [6] observed a reduction in photosynthetic parameters in oregano plants exposed to three drought stress regimes (100%, 75%, and 35% of FC). This phenomenon can be explained by disturbances in the biosynthesis of carbon skeletons, which are essential for producing photosynthetic assimilates and other key precursors under drought stress [22]. This disruption ultimately leads to reduced levels of photosynthetic pigments and lower overall plant yield. Additionally, reduced efficiency in carbon sequestration by the RuBisCO enzyme can result in altered metabolic pathways, causing the accumulation of lactate and ethanol. These changes are significant contributors to the reduction in photosynthetic pigments under severe water stress conditions [6]. Our results exhibited that mycorrhizal fungi treatment mitigated the adverse effects of drought stress and enhanced chlorophyll and carotenoid content. These fungi form a network of filaments that extend beyond the plant’s root system, significantly increasing its effective surface area for water uptake [30]. This improved water capture allows Marjoram plants to maintain higher leaf water content (RWC), a crucial factor for photosynthesis. Furthermore, the foliar application of SA improved the content of photosynthetic pigments. SA’s antioxidant properties help reduce the oxidation of chlorophyll molecules and regulate chlorophyll content [31]. This enhancement can result in a larger antenna size and maximized photosynthetic efficiency. Furthermore, Nazar [32] documented that SA mitigated damage to the photosynthetic apparatus under stress conditions by accumulating compatible solutes (such as proline), reducing toxic radical levels (ROS), and enhancing the activity of chlorophyll-synthesizing enzymes. Consequently, this leads to a decrease in chlorophyll degradation. Similar results were reported by Abdali [7] who investigated the SA combined with four other modifiers (containing amino acids, chitosan, ascorbic acid, and seaweed) on oregano plants under three levels of water deficit conditions (40, 60, and 75% of FC).

In the present study, drought stress increased proline accumulation (Fig. 4a). However, the SA foliar application and fungi inoculation led to significantly higher proline levels compared to drought stress alone. These findings are consistent with reports by Dianat [33] in *Lippia citriodora* L, La [34] in *Brassica rapa*, and Emrahi [6] in *Origanum vulgare*. Proline accumulation is a well-established response to drought stress. It acts as an osmolyte, helping plants maintain cell turgor pressure by regulating water balance within the cell [23]. Proline also functions as a potent scavenger of free radicals, mitigating the damaging effects of ROS generated under drought conditions [2]. Additionally, proline can serve as a source of energy and nitrogen when other resources become scarce [35]. Although proline is frequently considered a marker of stress, it is important to emphasize that the increased proline content observed under SA application and mycorrhizal fungi inoculation does not necessarily imply reduced exposure to drought stress. Instead, the elevated proline accumulation suggests an enhanced drought tolerance in these plants, with SA and fungi treatments playing a role in mitigating the adverse effects of drought. This increase in proline levels may reflect improved hydration and osmotic regulation, which in turn would support the plants’ ability to maintain turgor pressure and protect against oxidative damage, thereby facilitating an adaptive response to drought conditions. Notably, Nader [16] reported similar increases in proline levels following mycorrhizal fungi inoculation, despite the mitigation of drought stress. This further supports the notion that proline accumulation serves as a marker of stress adaptation rather than stress avoidance. Besides, the increased proline content under SA application may be due to the regulation of enzymes involved in proline metabolism, which play a crucial role in enhancing osmotic potential and cell turgor pressure, thereby improving plant adaptation to drought stress [7]. According to the results of this study, leaf proline content also increased under drought stress and mycorrhizal fungi inoculation. These fungi may enhance proline content under drought through several mechanisms: improved phosphorus uptake for proline biosynthesis enzymes [36], modulation of stress hormone signaling to promote proline gene expression [37], and priming of stress signaling pathways for a more robust proline response [37]. This combined effect of increased proline and improved water acquisition by fungi ultimately enhances plant drought tolerance by promoting cell integrity, protecting against ROS damage, and improving water management. Moreover, Moustakas [31] found that arbuscular mycorrhizal fungi inoculation of maize plants increased proline content under drought stress. In this study, as the intensity of drought stress increased, the soluble sugar content in plant leaves also rose, with the highest sugar content observed at the lowest irrigation level (D2) (Fig. 4b). It has been documented that under stress conditions, plants enhance the synthesis of compounds such as carbohydrates, which are integral to cell structure and growth, to maintain osmotic balance and improve water absorption from the root environment [38]. Drought stress also increases the activity of the enzyme alpha-amylase, leading to enhanced starch hydrolysis and, consequently, higher concentrations of soluble sugars [39]. Although the fungi application did not significantly affect soluble sugar levels, the numerical values were higher under this treatment. The fungi hyphal structures and their symbiotic relationship with root tissue cells enhance the plant roots’ absorption capacity and surface area. This improvement facilitates better nutrient transfer from the soil through mycorrhizal hyphae and stabilizes the soil under drought conditions [13]. Similar results were reported by Golubkina [13] who investigated fungi effects on yield and quality performances of *Artemisia dracunculus*, *Lavandula angustifolia*, and *Hyssopus officinalis*. Additionally, SA100 and SA300 foliar spraying of Marjoram plants increased the soluble sugar content in the leaves. SA application enhances the plant’s antioxidant efficiency, which can lead to increased chlorophyll content, improved photosynthesis, and higher carbon production. These processes not only strengthen the plant’s dry matter production but also enhance the accumulation of organic osmolytes, thereby improving osmotic regulation in the plant. Our results were in line with those of Abdali [7].

The defense mechanisms of plants against various biotic and abiotic stress factors are considerably enhanced by the activity of antioxidant enzymes. Under moisture deficiency, ROS accumulate in plant cells, leading to potential damage to cell membranes, lipids, nucleic acids, and carbohydrates and causing various metabolic changes [2]. To mitigate and detoxify the accumulation of ROS, plant cells deploy an enzymatic defense system that increases their tolerance to drought-induced stress. In this study, drought stress increased the levels of SOD, CAT, and POD, which enhanced the plant’s tolerance to drought stress (Fig. 5). During drought stress, a higher amount of free radicals is produced, prompting the plant to produce more antioxidant enzymes to counteract their harmful effects [6]. POD, present in both the cytosol and chloroplasts, can effectively eliminate H_2_O_2_. Therefore, the increased activity of this enzyme under drought stress is probably aimed at mitigating the negative effects of H_2_O_2_ [6]. CAT, an oxidoreductase located in peroxisomes, detoxifies H_2_O_2_ by breaking it down. Besides conferring drought resistance, CAT also plays a role in delaying senescence [7]. Besides, fungi application enhanced the activity of antioxidant enzymes, protecting the plant against ROS produced under stress conditions. One of the functions of POD is to participate in the cell’s defense system and detoxify ROS, thereby eliminating hydrogen peroxide produced by stress factors [13]. Similarly, Golubkina [13] reported that fungi treatment enhanced the antioxidant activity of *Artemisia dracunculus*, *Lavandula angustifolia*, and *Hyssopus officinalis* in the open-field experiment. In this study, SA300 foliar application also increased the activity of CAT, POD, and SOD. It appears that SA regulates the synthesis of antioxidant compounds during environmental stresses, enhancing the plant’s response to stress and contributing to drought tolerance [7]. These findings are inconsistent with the results of SA foliar application on Cowpea plants under drought stress [40]. Similarly, Azad [41] reported that SA foliar spraying of *Mentha pulegium* L. with 0.5 mM SA under drought stress increased the activity of SOD, APX, and CAT. This outcome can be linked to the SA’s protective balance between ROS generation and detoxification. Overall, SA functioned as a stimulant, increased the antioxidant enzymatic defense in studied plants under stress, and assisted in the development of plant tolerance to drought.

Furthermore, the factor analysis revealed distinct plant responses to drought stress, SA application, and mycorrhizal fungi, providing practical insights for managing plant growth under varying water deficit conditions (Fig. 6). Factor 1, which explains 56.60% of the variance under moderate drought (D1, SA100), is associated with key antioxidant enzymes, EL, proline, and soluble sugars, highlighting the importance of antioxidant defense and osmotic regulation in early stress response. This indicates that under mild stress conditions, enzymatic activities and osmolytes Like proline and soluble sugars are significantly affected, and targeting these biochemical pathways can enhance plant resilience. Factor 2 (2.41%), linked to severe drought (D2) and treatments with SA and fungi, emphasizes morphological traits such as plant height, Lateral branches, dry weight, and RWC, alongside CAT activity. This suggests that in more severe drought, maintaining structural integrity and water retention through SA and mycorrhizal fungi applications becomes critical for sustaining growth. Factor 3 (1.63%), related to non-stressed conditions (D0), associates mainly with photosynthetic parameters, reflecting their stability when water is not limiting. These findings underscore the practical significance of applying SA and mycorrhizal fungi to enhance both biochemical (POD, SOD, proline) and morphological traits, which can sustain plant growth and physiological function under drought stress. Besides, The correlation analysis revealed key relationships between plant dry weight and various physiological and morphological traits under drought conditions, providing valuable insights for optimizing plant growth (Fig. 7). A positive and significant correlation between plant dry weight and morphological and photosynthetic traits indicates that plants with greater morphological development and higher chlorophyll content tend to accumulate more biomass, reflecting healthier growth under both normal and stressed conditions. On the other hand, a negative and significant correlation was found between plant dry weight and stress-related parameters, including EL, antioxidant enzymes, and osmolytes. These results suggest that higher levels of these parameters are associated with reduced biomass, highlighting the detrimental effects of severe stress on plant growth. From a practical perspective, this implies that strategies aimed at reducing oxidative stress and stabilizing cellular integrity through treatments like SA and mycorrhizal fungi can positively influence plant biomass, supporting better growth and drought tolerance.

## Conclusion

This study demonstrated that the combined application of salicylic acid (SA) and mycorrhizal fungi (MF) significantly enhances marjoram’s drought tolerance by improving its morphological, physiological, and biochemical traits. While drought stress reduced plant height, Lateral branches, and chlorophyll content, the application of SA, particularly at 300 mg L^−1^, effectively mitigated these negative impacts. According to the hypothesis, although MF alone had limited effects, its combination with SA improved relative water content and reduced electrolyte leakage, indicating enhanced cell membrane stability under stress conditions. Additionally, the combined treatment increased proline accumulation, soluble sugars, and antioxidant enzyme activities, further boosting the plant’s defense mechanisms. The novelty of this study is associated with the integration of SA and MF as a dual approach to improving drought tolerance in marjoram, providing a unique strategy to address climate-induced water stress in crops. This approach is particularly important for sustainable agriculture, as it can provide a potential solution for improving crop resilience while reducing the dependency on synthetic chemicals. Furthermore, this study’s findings could be applied to marjoram production, a valuable herb with economic importance, especially in arid and semi-arid regions where water scarcity is a major challenge. Future research should focus on exploring the long-term effects of this combined treatment across different environmental conditions and marjoram varieties. In addition, assessing the potential of this approach for other crops could further expand its applicability. Investigating the underlying molecular mechanisms of the synergistic effects of SA and MF will provide deeper insights into their role in mitigating drought stress and could facilitate the development of more targeted strategies for improving crop resilience.

Data represent the mean of three replicates ± SE. Means with different letters have a significant difference (*P* ≥ 0.01). EL: electrolyte leakage; RWC: relative water content. D0: no drought stress or 90% of field capacity (FC); D1: drought stresses at 70% of FC; D2: drought stress at 35% of FC; SA0: no salicylic acid; SA100: salicylic acid treatment at 100 mg L-1; and SA300: salicylic acid treatment at 300 mg L-1. M0: No fungi inoculation; M1: with fungi inoculation.

## Supplementary Information

Below is the link to the electronic supplementary material.

Supplementary Material 1

## Data Availability

No datasets were generated or analysed during the current study.
