# Supplementary material for: Physiological and antioxidant responses of marjoram (Origanum Majorana L.) under drought stress mediated by Salicylic acid and mycorrhizal symbiosis
Source: BMC Plant Biol. 2025 Sep 30;25:1248. doi: 10.1186/s12870-025-07225-y (PMC12486875; doi:10.1186/s12870-025-07225-y)
Supplement: Supplementary file 1 — Supplementary Material 1 [file 12870_2025_7225_MOESM1_ESM.docx]

**Physiological and antioxidant responses of Marjoram (*Origanum majorana* L.) under drought stress mediated by salicylic acid and mycorrhizal symbiosis**

Babak Modara1, Mohammad Mehdi Rahimi 1*, Moslem Abdipoor2, Mehdi Hoseinifarahi3

1 Department of Agrotechnology, Yasuj Branch, Islamic Azad University, Yasuj, Iran.

2 Kohgiluyeh and Boyerahmad Agricultural and Natural Resources Research and Education Center, Agricultural Research, Education and Extension Organization (AREEO), Yasuj, Iran.

3 Department of Horticultural Science, Yasuj Branch, Islamic Azad University, Yasuj, Iran

*Corresponding author: Email: mohamadmehdirahimi9@gmail.com

**Table S1.** Soil physiochemical analysis of study area (2023-2024)

|  |  |  | **Soil** |  |  |  |
| --- | --- | --- | --- | --- | --- | --- |
| **K (ppm)** | **P (ppm)** | **N (%)** | **C (%)** | **EC dsm^-1^** | **pH** | **Soil texture**  **2023** |
| 205 | 5.9 | 0.044 | 0.91 | 0.98 | 7.73 | Clay loam |
|  |  |  |  |  |  |  |
| **K (ppm)** | **P (ppm)** | **N (%)** | **C (%)** | **EC dsm^-1^** | **pH** | **Soil texture**  **2024** |
| 195 | 6.5 | 0.085 | 0.83 | 0.68 | 7.45 | Clay loam |

**Table S2.** Analysis of variance of Marjoram morphological parameters

| SOV | df | Height | Dry weight | Lateral branch | Electrolyte leakage | Relative water content |
| --- | --- | --- | --- | --- | --- | --- |
| Year | 1 | 81.38** | 821139.60** | 90.07** | 72.72 | 14753.62** |
| Year*block | 4 | 35.47** | 292685.38** | 21.12 | 7.97 | 28.70 |
| D | 2 | 429.09** | 8095556.88** | 1725.83** | 5840.35** | 1935.73** |
| Year*D | 2 | 1.35 | 19355.06 | 2.93 | 2.21 | 229.40** |
| Error D | 8 | 2.26 | 19138.81 | 6.52 | 19.84 | 17.34 |
| SA | 2 | 295.47** | 4878353.63** | 963.03** | 493.50** | 684.68** |
| D*SA | 4 | 63.48** | 111793.82** | 96.04** | 148.08** | 13.94 |
| Year*SA | 2 | 0.02 | 710.78 | 0.21 | 0.77 | 54.92 |
| Year*D*SA | 4 | 0.17 | 2008.46 | 0.12 | 0.30 | 25.10 |
| M | 1 | 104.59** | 1004381.95** | 149.12** | 985.85** | 170.45** |
| D*M | 2 | 2.10 | 64647.56 | 27.56 | 113.95 | 4.54 |
| SA*M | 2 | 2.18 | 23166.44 | 84.91** | 203.79** | 57.64 |
| D*SA*M | 4 | 1.81 | 134311.95** | 54.40** | 98.95** | 23.00** |
| Year*M | 1 | 0.17 | 3083.20 | 0.44 | 0.61 | 12.70 |
| Year*SA*M | 2 | 0.14 | 578.82 | 0.06 | 0.06 | 28.44 |
| Year*D*M | 2 | 0.56 | 6210.98 | 0.57 | 0.37 | 15.06 |
| Year*D*SA*M | 4 | 0.19 | 1465.37 | 0.24 | 0.29 | 25.78 |
| Error | 60 | 4.26 | 26253.49 | 9.93 | 22.58 | 24.50 |
| CV |  | 5.57 | 4.25 | 7.94 | 13.14 | 7.33 |

**: significant in 1%, *” significant in 5%.

**Table S3.** Analysis of variance of Marjoram photosynthetic and biochemical parameters

| SOV | df | cola | colb | totalcol | cartonoied | POD | SOD | CAT | prolin | Soluble sugar |
| --- | --- | --- | --- | --- | --- | --- | --- | --- | --- | --- |
| Year | 1 | 0.0289** | 0.0433** | 0.1425** | 0.0011** | 0.1019** | 26879.03** | 0.0195** | 5.35** | 6151.74** |
| Year*block | 4 | 0.0003 | 0.0003 | 0.0002 | 0.0001 | 0.0001 | 4.10 | 0.0001 | 0.30 | 5.48 |
| D | 2 | 0.3423** | 0.0192** | 0.4113** | 0.0048** | 0.0621** | 2930.21** | 0.0727** | 292.14** | 2817.63** |
| Year*D | 2 | 0.0028** | 0.0012 | 0.0019 | 0.0000 | 0.0049** | 140.33** | 0.0097** | 12.80** | 89.22** |
| Error D | 8 | 0.0005 | 0.0006 | 0.0010 | 0.0001 | 0.0008 | 3.95 | 0.0001 | 0.73 | 12.20 |
| SA | 2 | 0.0558** | 0.0055** | 0.0270** | 0.0014** | 0.0071** | 242.33** | 0.0131** | 71.95** | 281.80** |
| D*SA | 4 | 0.0059** | 0.0042** | 0.0023 | 0.0010** | 0.0021** | 167.78** | 0.0070** | 4.59** | 119.97** |
| Year*SA | 2 | 0.0027** | 0.0003 | 0.0015 | 0.0000 | 0.0006 | 11.61 | 0.0017** | 1.86 | 12.63 |
| Year*D*SA | 4 | 0.0022** | 0.0003 | 0.0023 | 0.0000 | 0.0002 | 8.04 | 0.0009** | 1.03 | 39.20** |
| M | 1 | 0.0065** | 0.0041** | 0.0003 | 0.0010** | 0.0001 | 226.71** | 0.0051** | 46.21** | 111.98** |
| D*M | 2 | 0.0024** | 0.0014** | 0.0047** | 0.0003 | 0.0026** | 148.49** | 0.0017** | 1.25 | 5.57 |
| SA*M | 2 | 0.0033** | 0.0094** | 0.0096** | 0.0023** | 0.0021 | 97.63** | 0.0005** | 2.22 | 3.48 |
| D*SA*M | 4 | 0.0034** | 0.0009 | 0.0069** | 0.0002 | 0.0002 | 54.12** | 0.0018** | 1.50 | 3.87 |
| Year*M | 1 | 0.0001** | 0.0003 | 0.0000 | 0.0000 | 0.0000 | 10.86 | 0.0007** | 0.18 | 0.17 |
| Year*SA*M | 2 | 0.0016** | 0.0006 | 0.0016 | 0.0000 | 0.0002 | 4.68 | 0.0001 | 0.62 | 6.04 |
| Year*D*M | 2 | 0.0019** | 0.0001 | 0.0024 | 0.0000 | 0.0002 | 7.11 | 0.0002 | 1.49 | 2.20 |
| Year*D*SA*M | 4 | 0.0035** | 0.0001 | 0.0041** | 0.0000 | 0.0000 | 2.59 | 0.0002 | 1.90 | 7.73 |
| Error | 60 | 0.0006 | 0.0008 | 0.0011 | 0.0002 | 0.0009 | 11.81841 | 0.00011208 | 0.8382096 | 22.44369 |
| CV |  | 5.9200 | 29.95 | 6.45 | 29.15 | 25.98 | 4.76 | 28.73 | 4.71 | 5.38 |

**: significant in 1%, *” significant in 5%. Col a: Chlorophyll a; Col b: Chlorophyll b; Total col: Total chlorophyll; EL: Electrolyte leakage; POD: Peroxidase; CAT: Catalase; SOD: Super oxide dismutase
